# Studies on Morphological Evolution of Gravure-Printed ZnO Thin Films Induced by Low-Temperature Vapor Post-Treatment

**DOI:** 10.3390/nano14242006

**Published:** 2024-12-13

**Authors:** Giuliano Sico, Vincenzo Guarino, Carmela Borriello, Maria Montanino

**Affiliations:** 1 Italian National Agency for New Technologies, Energy and Sustainable Economic Development (ENEA), Portici Research Centre, P.le E. Fermi 1, Portici, 80055 Naples, Italy; carmela.borriello@enea.it (C.B.); maria.montanino@enea.it (M.M.); 2 Institute of Polymers, Composites and Biomaterials, National Research Council of Italy, Mostra d’Oltremare Pad. 20, V.le J.F. Kennedy 54, 80125 Naples, Italy

**Keywords:** zinc oxide, gravure printing, acetic acid, pressureless, nanojunctions, chemical sintering, grain rotation, nanoparticles coalescence, nanostructured networks, porous film

## Abstract

In recent years, the morphology control of semiconductor nanomaterials has been attracting increasing attention toward maximizing their functional properties and reaching their end use in real-world devices. However, the development of easy and cost-effective methods for preparing large-scale patterned semiconductor structures on flexible temperature-sensitive substrates remains ever in demand. In this study, vapor post-treatment (VPT) is investigated as a potential, simple and low-cost post-preparative method to morphologically modify gravure-printed zinc oxide (ZnO) nanoparticulate thin films at low temperatures. Exposing nanoparticles (NPs) to acidic vapor solution, spontaneous restructuring pathways are observed as a consequence of NPs tending to reduce their high interfacial energy. Depending on the imposed environmental conditions during the treatment (e.g., temperature, vapor composition), various ZnO thin-film morphologies are produced, from dense to porous ones, as a result of the activation and interplay of different spontaneous interface elimination mechanisms, including dissolution–precipitation, grain boundary migration and grain rotation–coalescence. The influence of VPT on structural/optical properties has been examined via XRD, UV–visible and photoluminescence measurements. Controlling NP junctions and network nanoporosity, VPT appears as promising cost-effective, low-temperature and pressureless post-preparative platform for preparing supported ZnO NP-based films with improved connectivity and mechanical stability, favoring their practical use and integration in flexible devices.

## 1. Introduction

In recent years, the morphology engineering of semiconductor nanomaterials has become a research area of increasing interest for controlling their functional properties and promoting their practical use in several applications [1,2]. In fact, such nanomaterials exhibit different physical–chemical properties compared to those of their bulk counterparts and distinctly size-dependent behavior, essentially due to their high surface-to-volume ratio [3]. This last feature enables a special sensitivity of these materials to external stimuli, which makes them particularly functional for several potential applications in many fields like energy, information, environment, microelectronics, biology and medicine [4,5,6,7]. Since the properties of semiconductor nanomaterials critically depend on their morphology, the possibility of engineering nanostructures for specific applications has become one of the most desirable research goals [8,9]. However, despite the exceptional properties of nanostructures, their use in single form is not feasible in real-world devices that would require greater area coverage to produce appreciable signals. Therefore, efforts are being made to develop methods for preparing nanostructured arrays which can combine high-performance single nanoscale building blocks in connected networks.

Among semiconductors, zinc oxide (ZnO) has probably the richest family of nanostructures and it is considered one of the most promising active materials for various cost-effective applications in optics, catalysis, gas sensors, energy generator, photovoltaics, electronics and biomedicine [6,10,11,12,13]. Over the past decade, several methods have been proposed to prepare different ZnO nanostructures, such as vapor phase transport, metal–organic chemical vapor deposition, chemical vapor deposition, thermal evaporation, pulsar laser deposition, electrodeposition, chemical bath deposition, and solvothermal and hydrothermal growth [14,15,16]. Many properties of the produced ZnO nanostructure depend on the preparative method used. Despite the reached material characteristics, most of the listed synthetic methods have poor industrial perspective for nanostructures fabrication, involving many steps or complex procedures, costly equipment, low throughput, high vacuum, high temperatures and low nanostructure uniformity [17,18,19]. In contrast, the solution-based techniques are to date the most used routes for growing ZnO nanostructures thanks to their low cost and simplicity [18]. On the other hand, such methods require long preparation times, and a high post-annealing temperature is often required to improve the crystallinity [17,20,21,22,23,24]. The high temperatures associated with the synthesis or post-thermal routes limit the choice of substrate to be used for ZnO nanostructuring, thus excluding flexible polymeric substrates (<150 °C). A further issue of such methods lies in the additional step required for preparing patterned nanostructures as required by many applications, which typically involves expensive preceding preparation of the seed layers or complex subsequent processes like photolithography and etching [25,26]. For all these reasons, the development of alternative low-temperature, practical and cost-effective synthetic strategies for preparing large-scale surface-patterned ZnO nanostructures is highly desirable [17,26,27].

In this regard, the use of crystalline nanoparticle (NP) systems can offer important advantages in low-temperature processing, since their synthesis can be performed separately from their deposition [28]. Therefore, an approach for an effective nanostructuring procedure can be found by associating two preparation methods, which share ease, low-temperature and low-cost characteristics, for distinguishing one step dedicated to the nanocrystal deposition and patterning and one subsequent step for their nanostructuring. Regarding the first step, low-temperature thin-film solution-based technology like high-throughput printing processes appear very attractive in terms of industrial scalability for producing patterned ceramic thin films on plastics, since they have been proven to be low-cost, fast and efficient techniques for deposition and patterning at the same time [29]. Among the printing techniques, the roll-to-roll (R2R) gravure is considered the most promising large-scale candidate, since it combines high-resolution patterning (<2 µm) with a high printing speed (up to 600 m min^−1^) and was recently demonstrated to be able to produce high-quality nanoparticulate ZnO thin films on plastics [30,31,32,33,34]. Regarding the post-preparative nanostructuring step, opportunities may come from the intrinsic metastability of the NP systems [35,36]. In fact, NP thin films possess a large internal surface which can be exploited for further modifications [37]. In the case of ZnO NPs, water molecules are typically adsorbed to their surfaces at ambient conditions for minimizing their high free energy in most practical applications [38,39,40]. Such adsorbed layers collectively constitute an energy barrier that stabilizes the NPs preserving their separation [41,42]. Once the surface chemistry of the stabilized NPs is changed, the spontaneous morphological modifications of the NP system may become possible, due to the natural tendency to reduce their free energy by interface elimination mechanisms [43].

Here, vapor post-treatment (VPT) is proposed as a potential, simple and low-cost post-preparative method to prepare ZnO thin-film nanostructured networks at low temperatures. In particular, ZnO NP thin films gravure-printed on flexible substrate were subjected to variable low-temperature vapor expositions through a two-step process for studying the possible morphological film evolution. A thermo-kinetic phenomenological approach was used to interpret the obtained results, describing the possible mechanisms underlying the observed morphologies. Varying the extent, the atmosphere composition and/or the temperature of the steps, final film morphology changes as a result of different spontaneous processes, including dissolution–precipitation, grain boundary migration and grain rotation–coalescence, as shown by some examples. Structural and optical properties of the treated films were also examined and discussed. Possible future opportunities were also sketched.

## 2. Materials and Methods

Undoped ZnO NP colloidal suspension (by Sigma-Aldrich Co., St. Louis, MI, USA) was deposited in thickness of 235 ± 10 nm onto polyethylenenaphthalate (PEN) film (Teonex^®^ Q65FA, DuPont Teijin Films, Chester, VA, USA) having a thickness of 125 µm (see Figure S1) and aluminum foil (Sigma-Aldrich Co.) having a thickness of 15 ± 1 µm by using a lab-scale gravure printer (IGT G1-5, Almere, The Netherlands) having a printing cylinder width of 5 cm; details of the experimental printing process were reported elsewhere [33]. All the prints were performed in air at room temperature. The thickness and the surface roughness of the dried printed layers was investigated by interferometry-based optical profilometer (Talysurf CCI HD, Taylor Hobson, Leicester, UK) (see Figure S2).

The performed VPTs comprised two steps. In the first one, the ZnO NP printed samples were exposed to the vapor of 1 M acetic acid aqueous solutions in a closed oven (VT 6025, Heraeus, Hanau, Germany) at 50 °C for removing the passivating hydroxylated layer of the NPs and, in some cases, for inducing chemical sintering. After the acidic step, distilled water was introduced in place of the acidic solution performing isothermal heating steps, as listed in Table 1.

The microstructural evolution of the treated samples was observed by field emission scanning electron microscopy (FESEM) (QuantaFEG200, Eindhoven, The Netherlands), after sputter-coating with gold–palladium, and by X-ray diffraction (XRD) (X’Pert MDP DY872 X-ray diffractometer, Malvern Panalytical Ltd., Malvern, UK), while their optical properties were characterized by performing UV–visible transmission (by Lambda 900, Perkin Elmer, Waltham, MA, USA) and room-temperature photoluminescence (PL) measurements (by Fluorolog 3, Horiba Jobin Instrument, Kyoto, Japan).

## 3. Results and Discussion

### 3.1. NP System Morphological Modifications Overview

From a classical thermodynamic perspective, a spontaneous morphological transformation of the NP system can occur if its total free energy can decrease toward a more stable state [44]. Possible routes for this reduction can include interface elimination processes, which are accomplished by matter transport mechanisms caused by gradients of one or more thermodynamic variables [45]. Since such thermodynamic driving forces can be of multiple nature (chemical potential gradients, temperature gradients, stress gradients and electric fields), different transport mechanisms, each having its rate, can be activated or be prevalent under a particular set of conditions. Depending on the degree of the system disequilibrium, different morphological rearrangement paths can occur as a result of the combination of thermodynamic and kinetic effects [46,47]. In particular, the combinations of the interfacial energies and specific transport coefficients that maximizes the energy reduction rate will provide the pathways for the system evolution [48]. During these paths, several phenomena having different lengths and time scales can take place and the system can evolve passing through possible intermediate states, in which it can be eventually frozen by imposing kinetic barriers [44].

In general, the energy of a particle depends on its size, shape and stress, and on the external environment [40]. Since the NP has large surface-to-volume ratio, also presenting larger crystal defects than bulk particle, it tends to have high reactivity, so NPs can be very sensitive to external stimuli or chemical environmental conditions [49,50,51,52,53,54]. The associated key quantity is the surface free energy, defined as the excess energy with respect to the bulk solid [55,56]. In a nanocrystal, such energy can be represented as the sum of many terms for each exposed crystallographic surface [57,58]. At first approximation, such quantity is proportional to the surface enthalpy, which refers to the energy of the surface as clean, i.e., an anhydrous surface [36,45,59].

In most practical cases, the surface adsorption of environmental contaminants on NP occurs to minimize the free energy state (stabilizing NP), changing the surface chemistry and consequently modifying the clean surface energy [38,39,60]. Therefore, in the case of an NP system embedded in a medium containing adsorbable components, interfacial energy, including the surface term related to the solid–vapor interface and grain boundaries (GBs) related to the solid–solid interface, has to be used [35,48]. Consequently, the interfacial energy is strongly variable depending on the composition of the surrounding medium which sets the potential [40]. If the surface chemistry of the stabilized NPs is changed, spontaneous mechanisms for lowering the high energy of the NPs can be made kinetically accessible under the corresponding thermodynamic conditions, also involving the morphological change in the whole NP system. For such evolution, mass transport mechanisms and/or the integration of the crystal building units have to be able to take place [45]. Depending on the occurrence of appropriate conditions in which the NP instability is activated and then driven, various morphological modification paths can be taken by the NP system, resulting from the interplay between thermodynamics (energetics stability) and kinetics (reactions and transport process rates) [45,47].

In this work, some of these possible rearrangement paths caused by the VPTs of gravure-printed ZnO NP thin films are shown and discussed as follows.

### 3.2. Acidic Vapor Post-Treatment for Activating ZnO NP Film Instability

**Figure 1:**
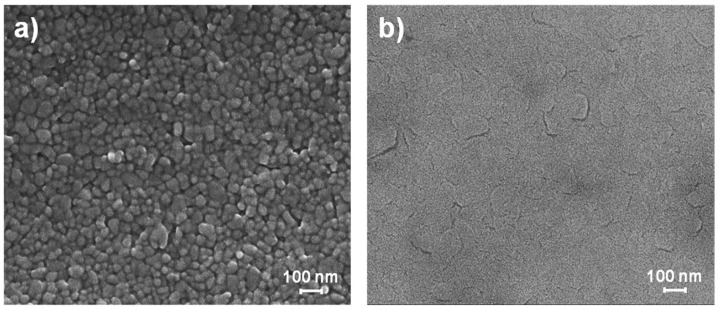
As-printed (untreated) ZnO NP film exposed to the vapor of a 1 M acetic acid aqueous solution in a closed oven at 50 °C: t = 0 (**a**); t > 150 min (**b**).

In a natural environment, ZnO NPs are typically heavily hydrated, due to the partial dissociative adsorption of water on their free surfaces at ambient conditions [35,39,40,41,61,62]. The covering hydroxylated layer passivates the high-reactivity surfaces giving stability to the NPs [41,63]. Setting appropriate conditions for removing such passivating layer can activate spontaneous mechanisms for lowering the NP high-energy state, resulting in possible morphological transformations at different scales. In this regard, recently, acidic vapor annealing was demonstrated to significantly affect the morphology of a ZnO NP film, inducing its spontaneous densification via a dissolution–precipitation mechanism [64]. Accordingly, when the as-printed ZnO NP film having a thickness of 235 ± 10 nm (see Figure 1a) is exposed to the vapor of a 1 M acetic acid aqueous solution at 50 °C, a time t* exceeding 75 min was found to be necessary for altering passivation layers, starting a densification process. During this period (t < 75 min), various physical–chemical mechanisms take place under constant external conditions, namely adsorption, dissolution and diffusion processes. When exposed to the vapor, a spontaneous adsorption process takes place on the surfaces of the ZnO solid NPs for further decreasing their energy [65]. Due to the typical presence of hydroxyl groups on their surface, ZnO NPs have a naturally hydrophilic character, which promotes the physisorption of additional adsorbed molecules that condense on the hydrated interface, further decreasing the NP energy [38,66]. As a consequence, the adsorbed layer spreads over the NP surfaces and becomes thicker, eventually overlapping between adjacent particles, where the additional physisorbed molecules can behave like a bulk liquid solution phase [38,67]. The vaporization at a constant temperature of the liquid aqueous solution from a sufficient reservoir keeps the vapor concentration at a constant level. The low annealing temperature favors the adsorption process, providing a slow kinetic for reaching equilibrium. Within the forming interfacial liquid layer, partial protonation of the NP surfaces occurs influencing their chemical nature, strongly enhancing the ZnO solubility as opposed to what happens in pure water [68,69,70]. As a consequence, upon acid solution condensation, the interfacial chemical dissolution of the solid ZnO NPs takes place, thus generating a mass transfer between adjacent phases [71]. In particular, the surface region of the NPs features a relatively large amount of corner and edge atoms as well as defects which are preferential detachment sites for starting the etching process due to their higher chemical potential [52,57,72]. Depending on the conditions imposed by the pH, different species (ions, charged species, fragments, neutral molecules) begin to populate the interfacial liquid [69,73,74]. As the dissolution goes on, the concentration of the thin interfacial liquid layer rapidly increases reaching supersaturation of ZnO products which start to precipitate. At this point, here identified in t*, if all the external conditions are kept constant, an autocatalytic reaction coupling dissolution and precipitation phenomena is established within the interfacial fluid, and thus, the recrystallization occurs at precipitation [75]. This happens by homogeneously nucleating and layer-by-layer growing on the parent NP surface within the interfacial fluid for lowering their chemical potential gradient; as the dissolution–precipitation mechanism proceeds, bonding among adjacent particles can occur. As reported in a previous work [64], over time, the energy reduction in the NP system is realized by turning the thermodynamically high-chemical-potential convex free surface of the NPs into GBs of low-energy flattened particles, and sintering is finally occurred at t > 150 min and very low temperatures (e.g., 50 °C), without using pressure (see Figure 1b). Waiting for more time, the GBs will tend to migrate for further reducing the total free energy causing a continuous grain growth phenomenon.

As a result, depending on the exposure time to the acid solution at constant conditions, different degrees of liquid-assisted densification (and consequently morphological transformation) of the ZnO NP thin film may be achieved, from the first inter-particle neck formation up to chemical sintering.

### 3.3. Vapor Post-Treatments of ZnO NP Films Changing Environmental Conditions

From classical thermodynamic theory, a far-from-equilibrium system can become more sensitive to normally negligible factors close to equilibrium, which may trigger its possible morphological evolution. As a result, in case the external conditions are eventually changed during the acidic vapor post-treatment, new transformation relationships can be introduced within the unstable ZnO NP system that can compete with the modality already established.

**Figure 2:**
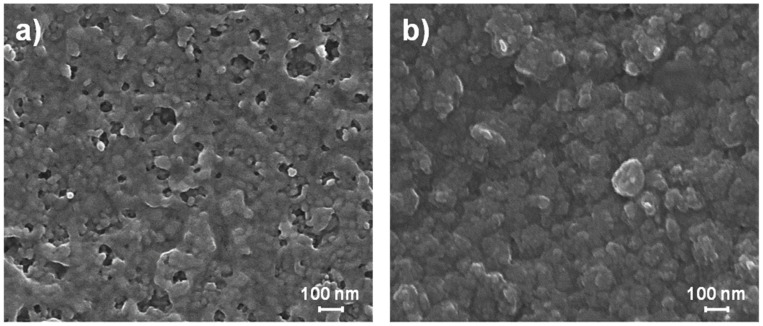
Microstructure at t = 150 min of as-printed ZnO NP films changing VPT environmental conditions at t > 75 min: by replacing the vaporizing acid solution with distilled water maintaining the temperature constant at 50 °C (**a**); by isothermal heating at 70 °C maintaining the acidic atmospheric composition (**b**).

So, once t* is exceeded, if only the atmosphere composition is changed, for instance, by replacing the vaporizing acid solution with distilled water, an incomplete sintering process is obtained, since the passivating hydroxylated layer is gradually restored as the acid vapor action fading over time (see Figure 2a). Meanwhile, if only the temperature is changed, for instance, by isothermal heating (at 70 °C), a spontaneous thermally activated grain coarsening process occurs during the liquid-assisted densification (see Figure 2b): on heating, a diffusive transfer of atoms (through the inter-particle liquid) from smaller to larger particles occurs for reducing the GB total area per volume [48,76,77]; typically, growth by coarsening tends toward nearly spherical morphologies, which are thermodynamically more stable due to the minimization of the overall surface energy [76,78].

**Figure 3:**
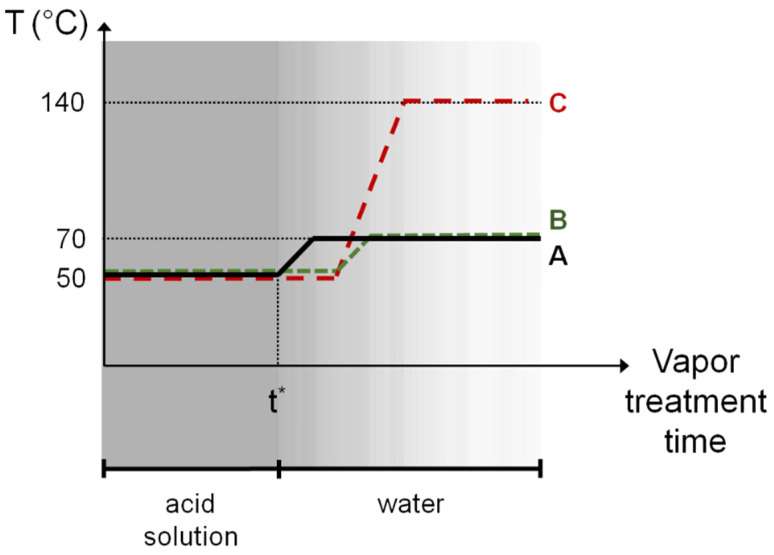
Schematic of the studied two-step vapor post-treatment profiles reported in the Materials and Methods section.

**Figure 4:**
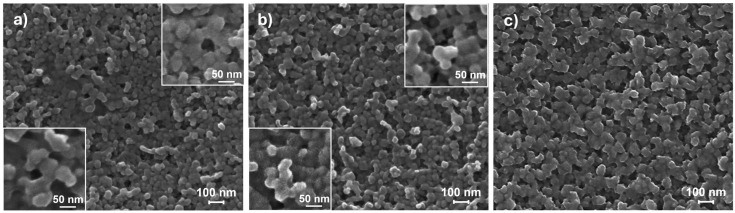
Microstructural evolution of as-printed ZnO NP film as a result of the treatment profile A: early stage of NP sliding and reorientation (**a**); intermediate stage of NP coalescence (**b**); porous leafage-like nanostructured network (**c**).

When the same imposed heating is coupled to the reduced dissolution effect by atmosphere composition change, as reported in Table 1 and depicted by treatments of Figure 3, a spontaneous restructuring of the ZnO NP thin film via a particle-based crystallization pathway can also take place, as described below. In Figure 4a–c, microstructural evolution images of the ZnO layer as a result of the treatment profile A are reported. Once the characteristic time t* for destabilizing the NP system is reached, the vaporizing acid solution is replaced by distilled water and the heating ramp is simultaneously started. As a consequence of the atmosphere change, the inter-particle condensed liquid solution gradually begins to reduce its acidity, mitigating the ZnO dissolution and thus slowing its reprecipitation. In such conditions, the imposed thermal gradient can add another mechanism for the structural rearrangement of the NP system, other than grains coarsening. In fact, a polycrystalline NP system can decrease its excessive energy stored in the GBs not only by decreasing the GB area, but also by decreasing the GB energy [49]. The latter is usually anisotropic, mainly depending on the misorientation angle between adjacent grains [79]. On heating, if there is a low degree of crystallographic misorientation, a spontaneous coalescence process of two crystalline grains is favored [76]. As a consequence, in addition to atomic diffusion at the particle surfaces and along the GBs, rigid NPs motion may also occur during heating, leading to lattice reorientation followed by the coalescence process [80]. Therefore, the less aggressive environmental condition can facilitate a reduction in the particle–particle crystallographic misorientation through a liquid-assisted misorientation energy-driven grain rotation mechanism before the coalescence process takes place. The still-present condensed inter-particle liquid phase favors weakly stuck nanograins sliding and rotation, so that NPs can more easily escape from their as-printed local potential minimum on heating, as the point-contact binding energies and thermal energy are of the same order of magnitude [80]. Since the GB energy depends on the crystallographic misorientation, its gradients drive particle rotations for lowering their free energy [81]. However, not all NPs can freely move, since some of them can be sterically hindered by other particles nearby; in this regard, grain rotations are expected to be mainly activated only for grains of relatively small size, since the grain rotation angular velocity is strongly grain-size dependent [49]. Consequently, where accessible, grain rotation–coalescence and fading dissolution–precipitation mechanisms can become simultaneously active during heating. Nevertheless, the NP reorientation is much faster than the other mechanisms [80], so that grains suddenly reorient in discontinuous steps upon heating caused by the torque for finding the nearest configuration corresponding to a lower local minimum of the grain-boundary energy [65,78,82,83]; in particular, faceted particles appear more prone to reorientation, since the crystallographic alignment can be satisfied on many points at a planar interface (see insets of Figure 4). Since anisotropic NP-NP interactions are non-prominent at the initial stage of this kind of attachment [84], no preferred orientation and neighbors are expected to emerge at this early stage [76]. As a result, a grain-reorientation-induced grain coalescence mechanism is established resulting in two distinct stages: a fast coordinated reorientation of neighboring grains in which particles convert into agglomerate (see inset of Figure 4a), followed by grains coalescence for eliminating the common GB between them (see inset of Figure 4b), thus forming a single larger grain [49,80]; where the reorientation is hindered, the lattice mismatch opposes the grain coalescence [80]. Once primary aggregates are formed, exceeding a critical size for allowed movements, grain reorientation practically stops [40,65,85], while the slowing dissolution–precipitation mechanism continues its action. Over time, the decrease in the system free energy is realized by GB reduction through the complete NP coalescence in an anisotropic nanostructure having multiple branches, eventually bonding the extremities of neighboring nanostructures. When the water adsorption passivation layer is finally restored, the system regains stability and a porous three-dimensional leafage-like nanostructured network is finally formed (see Figure 4c).

**Figure 5:**
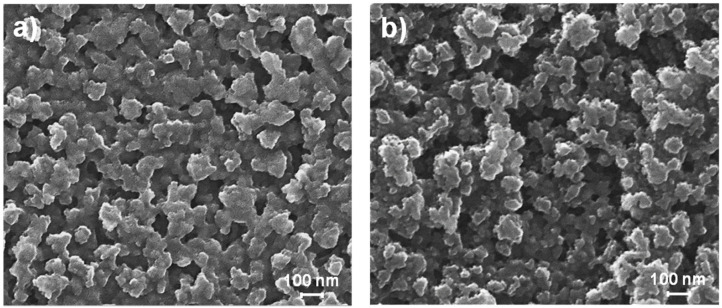
Microstructural evolution of as-printed ZnO NP films as a result of the treatment profile B (**a**) and C (**b**).

Depending on when the above-presented transients are introduced, the final morphology of the nanostructures can be changed accordingly. For example, if the heating step is started shortly after t* (see profile B in Figure 3), favoring an embryonic pre-sintering of the NPs, the particle self-assembly may also take place involving some small primary aggregates, so that the final nanostructured network will appear to have grown taller and with wider leaves (see Figure 5a). Furthermore, if the heating ramp is also performed at a higher temperature (for instance, 140 °C) after the short pre-sintering period (see profile C of Figure 3), a stepped gnawed structure is produced (see Figure 5b). In such a case, the liquid phase is rapidly evaporated, especially on the top of the ZnO clusters, while the acid concentration is still high in the vapor phase, so that an etching phenomenon may occur at high temperatures, generating jagged GBs.

**Figure 6:**
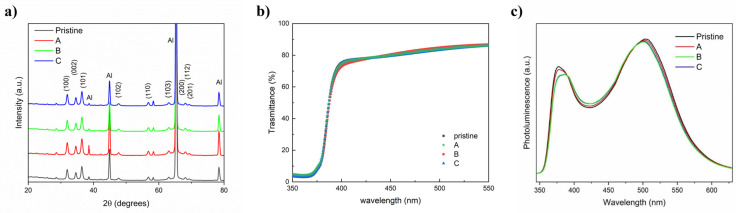
Structural and optical characterization of gravure-printed ZnO films on aluminum foil subjected to VPT profiles as reported in the Materials and Methods section: (**a**) XRD patterns; (**b**) optical transmittance; (**c**) photoluminescence spectra. Pristine refers to the as-printed (untreated) ZnO sample.

In order to investigate the effects of the observed morphological changes on the ZnO films characteristics, structural and optical spectroscopy measurements of the treated layers were carried out. In Figure 6, X-ray diffraction and the UV–visible transmission spectra of the representative treated ZnO films were reported. As can be seen, no significant changes associated with VPT were observed. In particular, the diffraction peaks of the starting ZnO NPs, compared with JCPDS data card (36–1451) [86], correspond to ZnO hexagonal wurtzite crystal structure that remained unaltered after treatments (Figure 6a). Moreover, VPT did not produce large agglomerates potentially acting as scattering centers, leaving the high optical quality of starting film unaffected (Figure 6b). The obtained optical band gap (Eg) of the ZnO films, estimated by Tauc’s relation [87], as reported in Figure S3, was constant, confirming no changes in crystallinity by the inter-atomic bond of the films with post-treatment [88]; a minimal deterioration of crystalline quality cannot be excluded, since a slight increase in the structural disorder degree, evaluated by the Urbach energy (Eu) [87], as reported in Figure S4, was obtained by VPT, as shown in Table 2. In this regard, as a very sensitive method for studying the structural degradation of crystalline thin films [21], room-temperature PL measurements at an excitation wavelength of 325 nm were also carried out (Figure 6c). As seen, the intensity of UV emission peak (~380 nm), considered strongly dependent on the film crystal quality [89], barely decreases only with VPT profile C, confirming a possible modest degradation of the crystalline quality; the observed minor red shift in such a peak may be quite compatible with the grain growth observed with VPT [89,90]. Similarly, the intensity of the broad visible emission peak (400–600 nm), believed to be related to various point defects [91], was effectively unchanged with VPT. The very slight blue-shift in such a peak may be attributed to the variation in the stoichiometry of the ZnO films with VPT [92].

Finally, a minimal increase in the surface roughness with VPT was also observed, in accordance with the SEM images (see Table 2).

### 3.4. Concluding Remarks and Opportunities

Spontaneous processes based on NP metastability can be used for inducing nanomaterial modifications as shown by VPTs. The associated key parameter is the interfacial energy relevant at the nanoscale for agglomerated and embedded systems. However, quantitatively capture interfacial energy changes to have a full thermodynamic description of the processing is a great experimental challenge, as a reliable method for obtaining interfacial energy data is still being studied, especially when dealing with NPs [36,48]. In this regard, recent advances in microcalorimetry combined with atomistic simulations may represent a useful tool for supporting the here-proposed thermo-kinetic picture in a future work.

**Figure 7:**
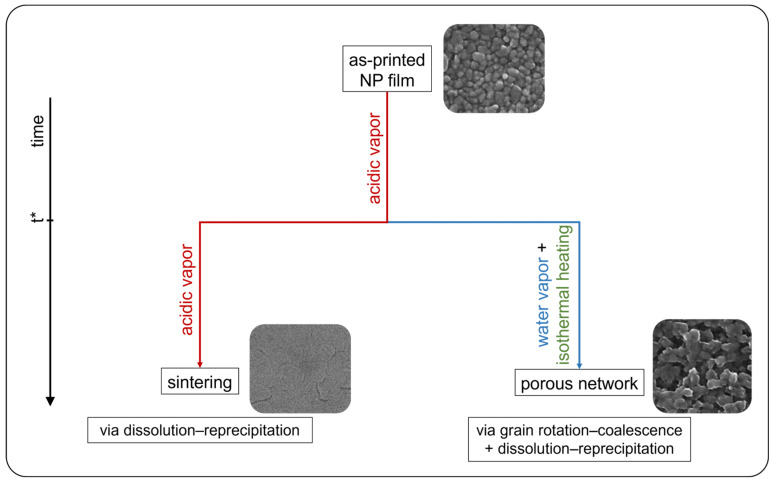
Diagram of the studied VPT processes.

The reported examples showed how VPT can represent a simple strategy for modifying nanoparticulate films. By appropriately controlling the environmental treatment conditions, VPT can activate NP film restructuring as a result of the combinations of interfacial energies and kinetic coefficients that deliver the fastest excess energy release rate [48]. In particular, as exemplified in the diagram in Figure 7, it was shown here that varying the surrounding medium composition and the temperature during the treatment can trigger various interface elimination mechanisms, responsible for the NP film morphology changing into sintered or porous structures. Uniform NP restructuring appears possible when VPT is applied to homogeneous and aggregate-free high-packing-density films such as those produced and here shown by gravure printing.

Since NP interfacial energy is a function of composition, many possible profiles/variants of the introduced method can be applied for reaching different ZnO-based film morphologies and properties by tuning interparticle nanojunctions and network nanoporosity degree and/or impurity introduction. In addition, the nanostructures obtained with the presented simple profiles may also be used as an intermediate process stage, upon which other treatments can be added depending on the desired application. A future in-depth analysis by means of TEM may help to more precisely capture the timing of the individual VPT stages.

Therefore, VPT, applied to patterned gravure-printed nanoparticulate films, can be considered as a versatile and scalable promising preparation strategy for pressureless low-temperature and low-cost fabrication on flexible substrates of variable density ZnO-based films, boosting their integration in our everyday life flexible devices. In particular, having no significant effects on the starting NPs crystallinity, VPT appears to be very interesting for preparing supported nanostructured networks for all those applications that intend to exploit ZnO NP special characteristics improving their connectivity and film mechanical stability at the same time, such as photocatalysts, photoanodes, photodetectors, sensors, membranes and solar applications [27,54,93,94,95,96,97,98,99], promoting the practical use of NPs in real-world devices. In this regard, VPT coupled with printing deposition technologies is expected to boost the use of NPs in advanced technologies applications at industrial scale [100], as long as the effectiveness of the method on a large scale and also for thick films is proven by additional work.

Finally, VPT appears in principle to be compatible with other materials having similar sensitivity to annealing atmosphere; nevertheless, preliminary tests must be carried out before adapting or extending the proposed method to other materials.

## 4. Conclusions

In this work, VPT was introduced as a potential, simple and low-cost platform to morphologically modify a ZnO nanoparticulate thin film at low temperatures. By exposing ZnO NP layers to acetic acid solution vapor, NP instability was triggered making possible spontaneous nanoscale restructuring as a consequence of the NP natural tendency to decrease their high interfacial energy. Depending on whether the imposed environmental conditions are kept constant or changed during the treatment, various thin-film morphologies, from dense to porous, can be produced as a result of different spontaneous interface elimination mechanisms, including dissolution–precipitation, grain boundary migration and grain rotation–coalescence. Such mechanisms were considered here on the basis of the experimental results and the literature. The performed VPTs were observed to have no significant influence on the crystalline and optical properties of the treated films. An effective and uniform VPT is obtained when applied to high-packing-density and high-quality NP films. As a result, VPT appears particularly advantageous when combined with high-quality printing thin-film-based technologies for rapid, low-cost and low-temperature patterned fabrication of supported ZnO NPs in nanostructured form, thus favoring ZnO-based nanostructure integration in flexible devices. These findings showed the high technological potential of the spontaneous processes for nanocrystals solid morphology manipulation, allowing new opportunities for simple and low-cost ceramic thin-film manufacturing, especially when pressure- and temperature-sensitive materials are involved.

## Figures and Tables

**Table 1:** Performed two-step vapor post-treatments on gravure-printed ZnO thin films.

Post-Treatment Profile Name	Vaporizing Solution	
	1st Step	2nd Step
A	1 M acetic acid aqueous solution at 50 °C for 75 min	Distilled water at 70 °C for 90 min
B	1 M acetic acid aqueous solution at 50 °C for 90 min	Distilled water at 70 °C for 90 min
C	1 M acetic acid aqueous solution at 50 °C for 90 min	Distilled water at 140 °C for 90 min

**Table 2:** Morphological and optical characteristics of the vapor post-treated gravure-printed ZnO films; pristine refers to the as-printed (untreated) ZnO sample.

Post-Treatment Profile Name	Surface Roughness [nm]	Optical Band Gap [eV]	Urbach Energy [meV]
pristine	15 ± 1	3.23	56.1
A	17 ± 1	3.22	60.9
B	21 ± 3	3.22	64.0
C	28 ± 4	3.22	64.2

## Data Availability

The raw data supporting the conclusions of this article will be made available by the authors on request.

## Supplementary Materials

The following supporting information can be downloaded at: https://www.mdpi.com/article/10.3390/nano14242006/s1, Figure S1: Example of gravure-printed ZnO film on PEN plastic substrate after VPT; Figure S2: Example of profile extraction for the thickness estimation of the as-printed ZnO sample by interferometry-based optical profilometer; Figure S3: Tauc plots for the estimation of the optical bandgap of gravure-printed ZnO films subjected to VPT profiles as reported in the Materials and Methods section: (a) as-printed (untreated sample); (b) profile A; (c) profile B; (c) profile C; Figure S4: Urbach plots for gravure-printed ZnO films subjected to VPT profiles as reported in the Materials and Methods section: (a) as-printed (untreated sample); (b) profile A; (c) profile B; (c) profile C;.
