# Supplementary material for: Studies on Morphological Evolution of Gravure-Printed ZnO Thin Films Induced by Low-Temperature Vapor Post-Treatment
Source: Nanomaterials (Basel). 2024 Dec 13;14(24):2006. doi: 10.3390/nano14242006 (PMC11728543; doi:10.3390/nano14242006)
Supplement: Supplementary file 1 [file nanomaterials-14-02006-s001.zip › nanomaterials-3312600-supplementary.pdf]

Article

Supplementary materials: Studies on Morphological Evolution of Gravure Printed ZnO Thin Films Induced by Low Temperature Vapor Post-Treatment

Giuliano Sico [1,*], Vincenzo Guarino [2,*], Carmela Borriello [1] and Maria Montanino [1]

[1] Italian National Agency for New Technologies, Energy and Sustainable Economic Development (ENEA), Portici Research Centre, P.le E. Fermi 1, 80055 Portici, Naples, Italy; carmela.borriello@enea.it; maria.montanino@enea.it

[2] Institute of Polymers, Composites and Biomaterials, National Research Council of Italy. Mostra d'Oltremare Pad. 20, V.le J.F. Kennedy 54, 80125, Naples, Italy.

* Correspondence: giuliano.sico@enea.it (G.S.); vincenzo.guarino@cnr.it (V.G.)

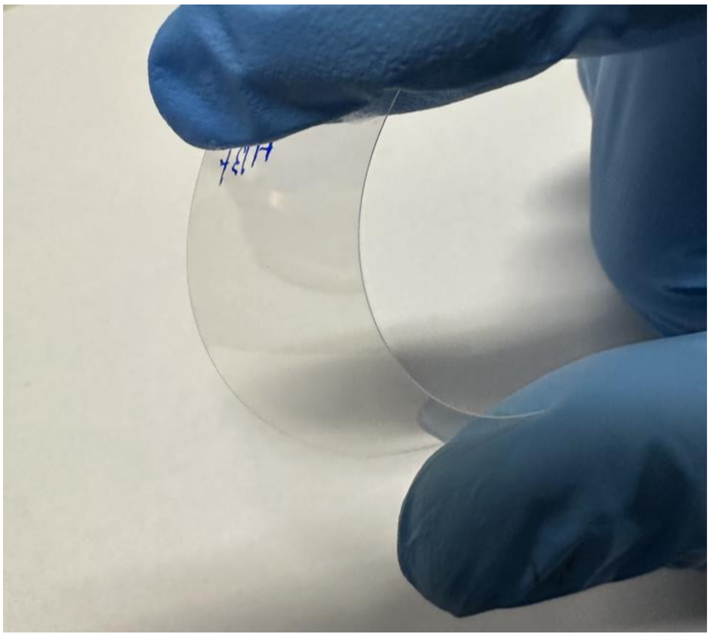

Figure S1. Example of gravure printed ZnO film on PEN plastic substrate after VPT.

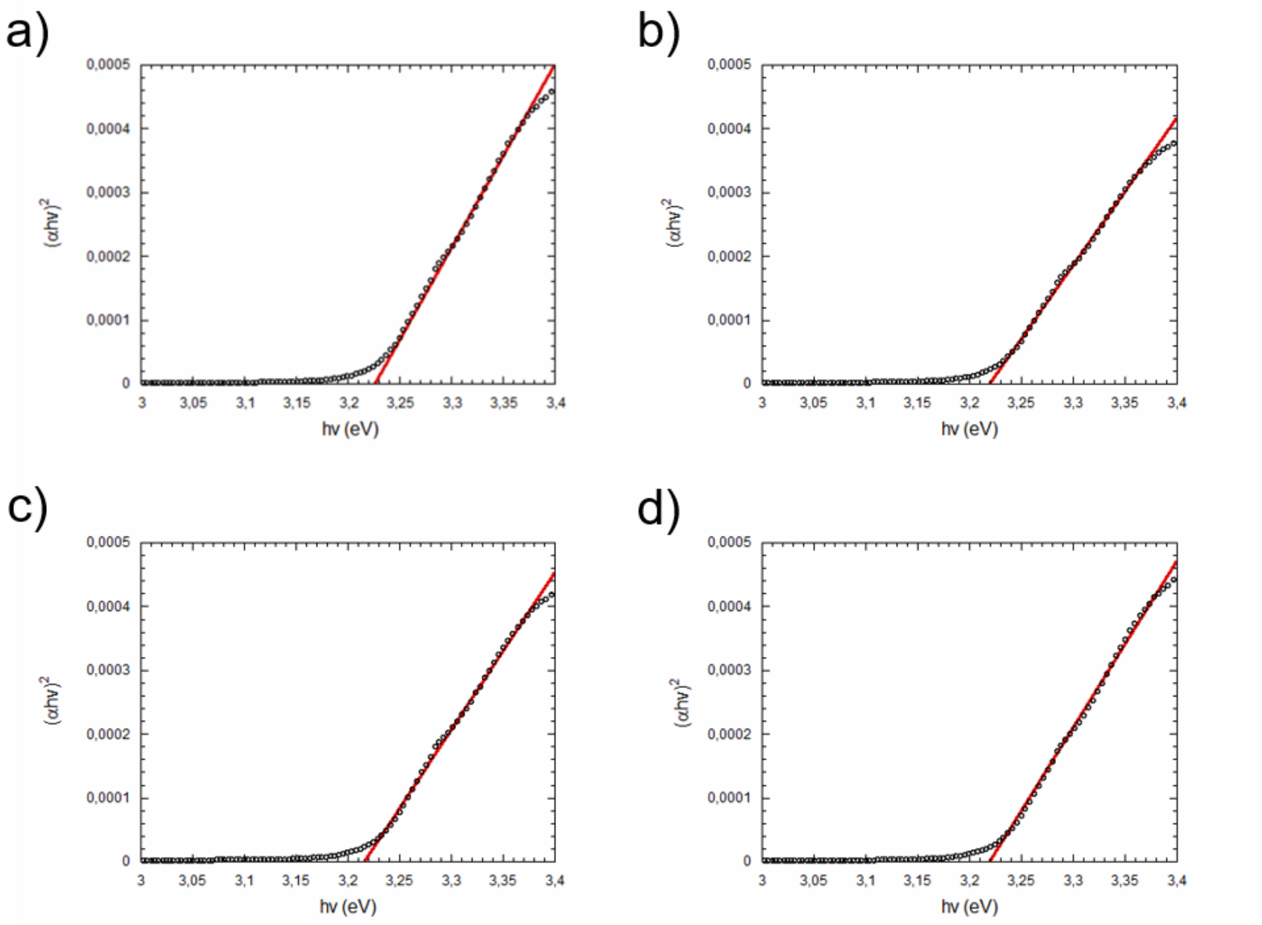

Figure S2. Tauc's plots for the estimation of the optical bandgap of gravure printed ZnO films subjected to VPT profiles as reported in Experimental section: a) as-printed (untreated sample); b) A profile; c) B profile; c) C profile.

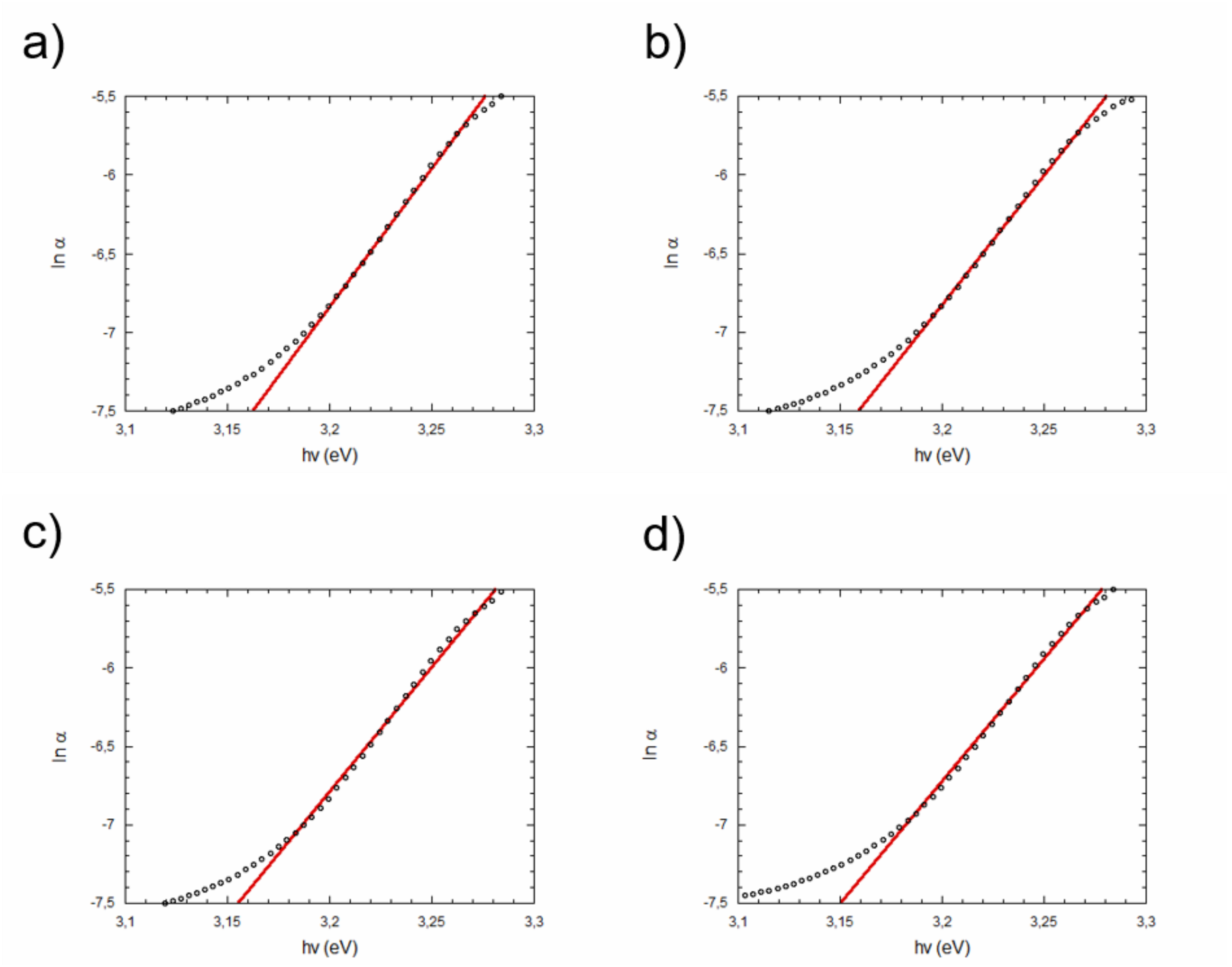

Figure S3. Urbach plots for gravure printed ZnO films subjected to VPT profiles as reported in Experimental section: a) as-printed (untreated sample); b) A profile; c) B profile; c) C profile.

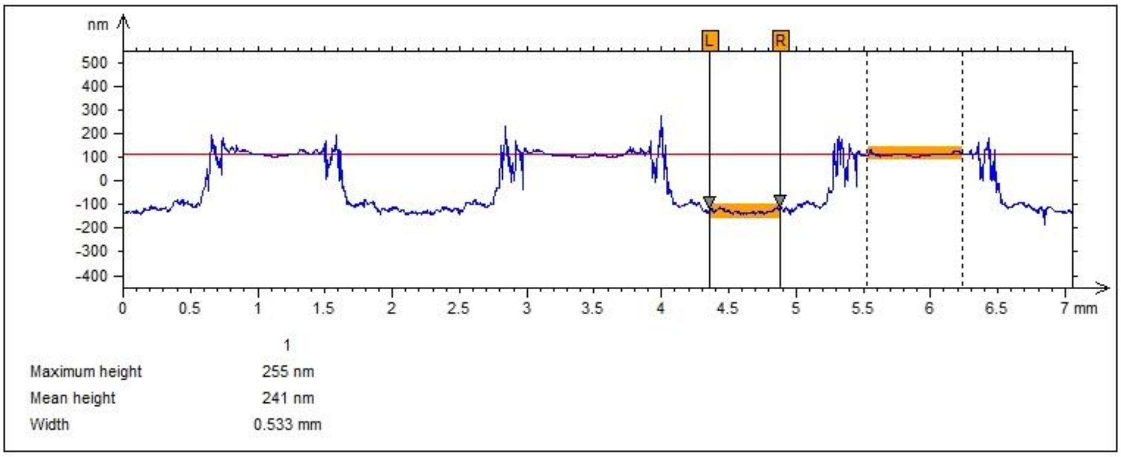

Figure S4. Example of profile extraction for the thickness estimation of the as-printed ZnO sample by interferometry-based optical profilometer.
